# No effect of 10-week training in click-based echolocation on auditory localization in people who are blind

**DOI:** 10.1007/s00221-021-06230-5

**Published:** 2021-10-05

**Authors:** Lore Thaler, Liam J. Norman

**Affiliations:** Department of Psychology, Durham University, Science Site, South Road, Durham, DH1 3LE UK

**Keywords:** Sensory learning, Audition, Neuroplasticity, Blindness, Cognition, Behaviour

## Abstract

What factors are important in the calibration of mental representations of auditory space? A substantial body of research investigating the audiospatial abilities of people who are blind has shown that visual experience might be an important factor for accurate performance in some audiospatial tasks. Yet, it has also been shown that long-term experience using click-based echolocation might play a similar role, with blind expert echolocators demonstrating auditory localization abilities that are superior to those of people who are blind and who do not use click-based echolocation by Vercillo et al. (Neuropsychologia 67: 35–40, 2015). Based on this hypothesis we might predict that training in click-based echolocation may lead to improvement in performance in auditory localization tasks in people who are blind. Here we investigated this hypothesis in a sample of 12 adult people who have been blind from birth. We did not find evidence for an improvement in performance in auditory localization after 10 weeks of training despite significant improvement in echolocation ability. It is possible that longer-term experience with click-based echolocation is required for effects to develop, or that other factors can explain the association between echolocation expertise and superior auditory localization. Considering the practical relevance of click-based echolocation for people who are visually impaired, future research should address these questions.

**Supplementary Information:**

The online version contains supplementary material available at 10.1007/s00221-021-06230-5.

## Introduction

There is a substantial body of research investigating how the brain adapts in the context of blindness (for reviews see Bavelier and Neville 2002; Burton 2003; Maidenbaum et al. 2014; Merabet and Pascual-Leone 2010; Noppeney 2007; Renier et al. 2014; Röder and Rösler 2004; Kupers and Ptito 2014). Yet, it is still an open question how visual experience affects people’s ability to generate and calibrate mental representations of their spatial surroundings. For example, with respect to behavioural performance in spatial hearing tasks, it has been found that people who are blind may show better performance as compared to people who are sighted, e.g., localization of sounds in the periphery (Battal et al. 2020; Röder et al. 1999; Voss et al. 2004), monoaural localization (Lessard et al. 1998), or perception of sound distance (Kolarik et al. 2013; Voss et al. 2004). People who are blind may also, however, show worse performance compared to people who are sighted, e.g., localizing vertical location of sounds (Zwiers et al. 2001; Lewald 2002; but also see Battal et al. 2020 who report superior performance) or judging relative spatial position between three successive sounds, also referred to as auditory spatial bisection (Gori et al. 2014). These findings would suggest that visual experience is essential for successful spatial calibration in the human brain.

It remains possible, however, that the role of vision in spatial calibration might be substituted by expertise in a non-visual sensory skill. One example of such a skill is echolocation, which is the ability to use reflected sound to get information about the environment. Even though echolocation is primarily associated with bats, it is by now well established that humans are also able to use it (Kolarik et al. 2014; Stroffregen and Pittenger 1995; Thaler and Goodale 2016). A distinction can be made between passive and active echolocation. For passive echolocation, the listener processes emissions and echoes where emissions have been made by sources other than the listener themselves, e.g., ambient sound fields, another person speaking, making mouth-clicks, etc. For active echolocation, echolocators make their own emissions and use echoes arising from those, e.g. echoes from one’s own mouth clicks, footsteps, cane taps, etc. Laboratory research has shown that echolocation using mouth-clicks, i.e., click-based echolocation, provides sensory advantages above and beyond passive echolocation via ambient sound fields, e.g. (Ekkel et al. 2017; Teng and Whitney 2011; Thaler et al. 2014) or active echolocation using footsteps or cane-taps (Kolarik et al. 2017; Thaler et al. 2019). It has also been shown that click-based echolocation provides real-life advantages for people who are blind in terms of their mobility, independence, and wellbeing (Norman et al. 2021; Thaler 2013).

Most relevant to the question of whether calibration of spatial representations can take place in the absence of visual experience, it has been found that people who are blind but who use click-based echolocation do not show performance deficits in auditory spatial bisection tasks (Tonelli et al. 2020; Vercillo et al. 2015). They also showed superior performance in tests measuring minimum audible angles, as compared to people who are sighted or to people who are blind and do not use click-based echolocation (Vercillo et al. 2015). Based on these findings it has been suggested that click-based echolocation may not only help in the calibration of auditory space for people who are blind, but that for spatial bisection, it might possibly even substitute for the role played by visual sensory calibration (Tonelli et al. 2020; Vercillo et al. 2015).

The current study investigated how blind people’s performance in auditory localization changes as a consequence of learning click-based echolocation. Based on the idea that click-based echolocation may help in the brain’s ability to calibrate auditory space, we might expect that training in click-based echolocation would improve performance in spatial bisection and minimum audible angle tasks in people who are blind.

In our study, 12 people who were blind were trained in click-based echolocation over the course of 10 weeks. Before and after training we also measured people’s ability to localize sound sources (Minimum Audible Angle Task) and to perform auditory spatial bisection (Spatial Bisection Task). Based on the idea that click-based echolocation might help calibration of auditory space we expected that training in click-based echolocation would lead to improved performance in both tasks. Our results, however, show no evidence for improvement after training echolocation for 10 weeks despite a clear improvement in echolocation ability.

## Methods

### Ethics statement

All Procedures followed the British Psychological Society code of practice and the World Medical Association’s Declaration of Helsinki. The experiment had received ethical approval from the Ethics Advisory Sub-Committee in the Department of Psychology at Durham University (Ref 14/13). All participants gave written informed consent to take part in this study. Participants received £10/hr to compensate them for their effort and time taking part.

### Data availability statement

Data are available as Supplementary Material S1.

Details of the training and training results are described in Norman et al (2021), but all raw data for training results are also contained in Supplementary Material S1. Briefly, all participants were trained to echolocate using mouth clicks over the course of 10 weeks (20 sessions, each between 2 and 3 h in length). Over the course of the training, people’s performance improved in three different echolocation tasks (size discrimination, orientation identification, virtual navigation) to a level that in most (but not all) cases matched performance demonstrated by experts. To summarise the improvements in performance, on average participants improved by 26% in orientation identification, 21% in size discrimination, and 22% in the ability to successfully navigate through a virtual echo-acoustic maze. These measures were calculated by taking the difference in accuracy between the first and final training sessions. Participants also reported positive effects of training on their mobility, wellbeing, and independence outside the lab (i.e., in their daily lives).

### Participants

Twelve blind participants (BCs; 6 males, 6 females) with no prior experience in click-based echolocation took part. The number of participants was determined by practical limitations, i.e., the availability of people who were blind to take part in the research, but we used power analysis with G*Power 3.1 (Faul et al. 2007) to confirm that we had the required sample size to detect a significant effect at alpha 0.05 and power of 0.95. We visually estimated the expected effect size based on the data provided in Vercillo et al. (2015) Fig. 4a and b for participants who were blind and not echolocating (*n* = 6) or echolocating (*n* = 3). Thus, we used the reported between-group differences (and within-group variances) to estimate changes that we might expect to see in our sample from pre to post-training tests. Based on Vercillo et al. (2015) data we estimated the expected effect size for spatial bisection and minimum audible angle thresholds to be 3.03 and 3.20, respectively, and the minimum required sample size to be 4. Thus, we had sufficient power in our study. Details of the sample have been described in Norman et al. (2021) but are also listed in Table 1 and described here. In our sample, all BCs had a cause of vision loss present from birth. All were diagnosed as legally blind in childhood, with only two official diagnoses at an age that might have coincided with onset of puberty, or may have been after onset of puberty (i.e., 13 years and 10 years; BC6 and BC2), but again with vision impairment having been present from birth. Thus, the majority of our participants are classified as early blind. With the exception of one blind participant (BC8, aged 72 years) who wore hearing aids to compensate for age-related hearing loss, all participants had normal hearing appropriate for their age group (ISO 7029:2017) assessed using pure tone audiometry (0.25, 0.5, 1, 2, 4, 8 kHz) (Interacoustics AD629, Interacoustics, Denmark; Hughson Westlake procedure). For purposes of testing, the participant with hearing aids did not wear their aids during any of the experimental testing sessions. All participants who had any residual vision were tested under blindfold.

**Table 1:** Details of participants who were blind. Unless otherwise stated, official diagnosis from birth/within first year of life

ID	Gender	Age	Degree of vision loss	Cause and age at onset of vision loss	Echolocation use prior to taking part
BC1	F	60	Total blindness in left eye; some peripheral vision in right eye	Stichler’s syndrome. Retinal sciasis, from birth with increasing severity	Some experience; very little regular use
BC2	M	54	Residual bright light perception	Retinitis pigmentosa. Official diagnosis age 10 years. Gradual sight loss from birth	Some experience; very little regular use
BC3	M	39	Residual bright light perception	Retinitis pigmentosa. Gradual sight loss from birth. Official diagnosis in early childhood (no exact age remembered but was known when commencing school, i.e., age 5 years)	None
BC4	M	46	Total blindness	Ocular albinism. Gradual sight loss from birth	Some experience; very little regular use
BC5	F	36	Bright Light detection	Unknown cause; from birth	None
BC6	M	37	Tunnel vision (< 5 deg) and decreased acuity (< 20/200) in both eyes	Retinitis pigmentosa. Gradual sight loss from birth. Official diagnosis age 13 years	None
BC7	M	48	Total blindness in left eye; residual bright light perception in right eye	Severe childhood glaucoma; 3 months old	None
BC8	F	72	Bright Light detection	Retinitis Pigmentosa. Gradual sight loss from birth. Official diagnosis in early childhood (no exact age remembered but was known when commencing school, i.e., age 5 years)	None
BC9	F	79	Some blurred foveal vision; prone to bleaching	Rod Cone Dystrophy. Birth	None
BC10	F	44	Total Blindness right eye; bright light detection left eye	Microphtalmia and Glaucoma; right eye enucleated aged 39 years	None
BC11	F	27	Left eye ca. 1 deg of foval vision left with reduced acuity (< 20/200); right eye bright light detection	Leber’s Amaurosis and Cataracts. Birth	None
BC12	M	38	Tunnel vision (< 2 deg) and decreased acuity (< 20/200) in both eyes	Retinitis Pigmentosa and other retinal pathology (unknown). Official diagnosis in early childhood (no exact age remembered but was known when commencing school, i.e., age 5 years)	None

### Apparatus and procedures

All testing took place in a sound-insulated and echo-acoustic dampened room (approx. 2.9 m × 4.2 m × 4.9 m) lined with foam wedges (cut-off frequency 315 Hz) in the department of psychology at Durham University. Tests were run with MATLAB R2018b (The Mathworks, Natick, MA) and modified functions from the Psychtoolbox library (Brainard 1997) on a laptop (Dell Latitude E7470; Intel Core i56300U CPU 2.40; 8 GB RAM; 64-bit Windows 7 Enterprise) with an external sound card (Creative Sound Blaster External Sound Card Model SB1240; Creative Technology Ltd., Creative Labs Ireland, Dublin, Ireland; 24 bit and 96 kHz). Stimuli were presented through headphones (Etymotic ER4B; Etymotic Research, Illinois, USA) with the highest peak intensity presented at 80 dB SPL. Any session lasted approx. 2 h, depending on how many and how long breaks participants wished to take.

### Spatial tasks (pre and post)

#### Sound stimuli

**Fig. 1:**
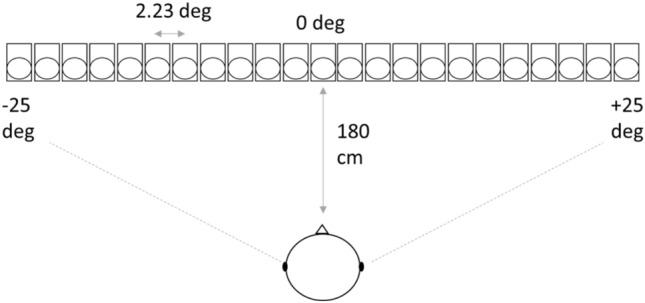
Illustration of the spatial arrangement used for the sound recording procedure ( adapted from Vercillo et al. 2015). Recordings were made using binaural microphones in the ears of a participant facing a loudspeaker at one of 23 different horizontal placements. The central placement (i.e. a horizontal offset of 0°) was positioned at a distance of 180 cm to the participant

The sounds we used were the exact same as those used by Vercillo et al (2015), as the authors had kindly shared their sound files with us. Details of the sounds and task are described in those reports, but they are summarised briefly here. The sound recordings were binaural recordings of 500-Hz 75-ms tones that were produced from loudspeakers at 23 different locations relative to the participant’s ears. The loudspeakers were facing the participant, in positions ranging from left to right (− 25° to + 25° of visual angle) relative to centre (0°). Figure 1 shows the spatial arrangement of the sound stimuli, as adapted from Fig. 1a in Vercillo et al. (2015).

#### Paradigm

##### Spatial bisection task

**Fig. 2:**
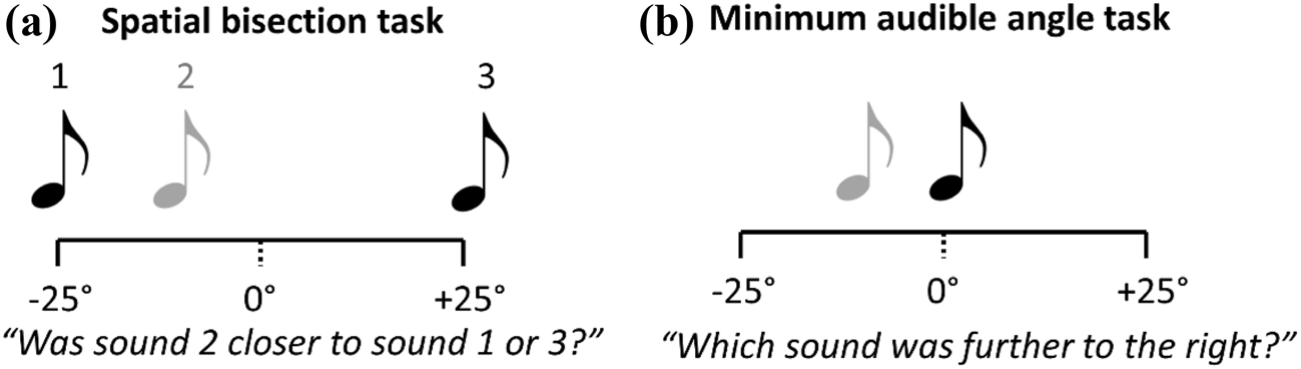
Illustration of the tasks used to measure aspects of spatial hearing. **A** and **B** show the spatial bisection task and minimum audible angle task, respectively. The black tones indicate sounds that were fixed on every trial, serving as reference stimuli. The grey tones indicate sounds that varied from trial to trial, serving as comparison stimuli. On each trial, the sounds in task A were played in a fixed order (1–3), whereas the sounds in task B were played in a randomly determined order. The tasks were replications of those reported in Vercillo et al. (2015)

This task replicated that used by Vercillo et al (2015). The sound recordings were used in a psychophysical task to measure participants’ ability to judge whether the second of three sounds was closer in space to the first or the third. Thus, this task requires participants to judge the relative location of a comparison sound with respect to two reference sounds, regardless of the participant’s own perceived location, i.e., they have to make an allocentric spatial judgment. On each trial, participants first heard the sound at the leftmost position (− 25°, reference sound 1) followed by another at one of the possible 23 locations ranging from − 25° to + 25° (the comparison sound), and then a final sound at the rightmost position (+ 25°, reference sound 2). Sounds were presented with an inter-stimulus interval of 500 ms. After hearing all three sounds, participants pressed one key to indicate that the second sound was closer in space to the first (leftmost) sound, or another key to indicate that the second sound was closer in space to the third (rightmost) sound. Figure 2A illustrates this task. Performance on feedback was not given. Participants completed 9 repetitions for each location of the comparison stimulus, giving a total of 207 trials. Before completing the task, participants completed a short practice session of 16 trials.

##### Minimum audible angle task

This task was also a replication of a task used by Vercillo et al (2015). The sound recordings were used in a psychophysical task to measure participants’ ability to judge which of two sounds was located further to the right of the participant’s egocentric midline, i.e., they have to make an egocentric spatial judgment. On each trial, participants heard the 0° sound (the reference sound) and another at one of the possible 23 locations ranging from − 25° to + 25° (the comparison sound). Sounds were presented with an inter-stimulus interval of 500 ms. Importantly, the two sounds were presented in a random order and after hearing both sounds, participants could press one key to indicate that the first sound was located more to the right, or another key to indicate that the second sound was located more to the right. Figure 2B illustrates this task. Feedback on performance on each trial was not given. Participants completed 9 repetitions for each location of the comparison stimulus, giving a total of 207 trials. Before completing the task, participants completed a short practice session of 16 trials.

### Data analysis

Response data were collated separately for each task (spatial bisection and minimum audible angle tasks) and session (pre-training and post-training). For each position of the comparison sound, the proportion of trials in which the participant indicated that the comparison sound was closer to the rightmost position was calculated. A cumulative normal distribution was then fit to these data using the Palamedes toolbox for Matlab (Prins and Kingdom 2018), yielding Point of Subjective Equality (PSE, given by the mean) and threshold (standard deviation). Threshold is a measure of sensitivity (lower values indicating greater sensitivity) and PSE is a measure of response bias (values closer to zero are less biased). These data were analysed with SPSS v26 using paired t-tests and estimation of Bayes Factors (null/alternative). A Bayes factor of one indicates that null and alternative are equally likely. A Bayes factor larger than one indicates that the data are in favour of the null hypothesis (i.e., no difference in performance between pre and post sessions). The normality of the difference between the pre- and post-training measures for each of the four variables (auditory spatial bisection threshold and PSE; minimum audible angle threshold and PSE) was assessed using a Shapiro–Wilk test. All of the tests returned a non-significant result (auditory spatial bisection threshold: *W*(12) = 0.885, *p* = 0.102; auditory spatial bisection PSE: *W*(12) = 0.927, *p* = 0.354; minimum audible angle threshold: *W*(12) = 0.862, *p* = 0.051; minimum audible angle PSE: *W*(12) = 0.965, *p* = 0.956), indicating that the assumption of normality is held. Furthermore, we used analysis of standardized residuals to assess linear model fit and to screen for potential outliers or extreme data points. We did not find evidence for concern or to suggest the removal of any participant’s data.

## Results

**Fig. 3:**
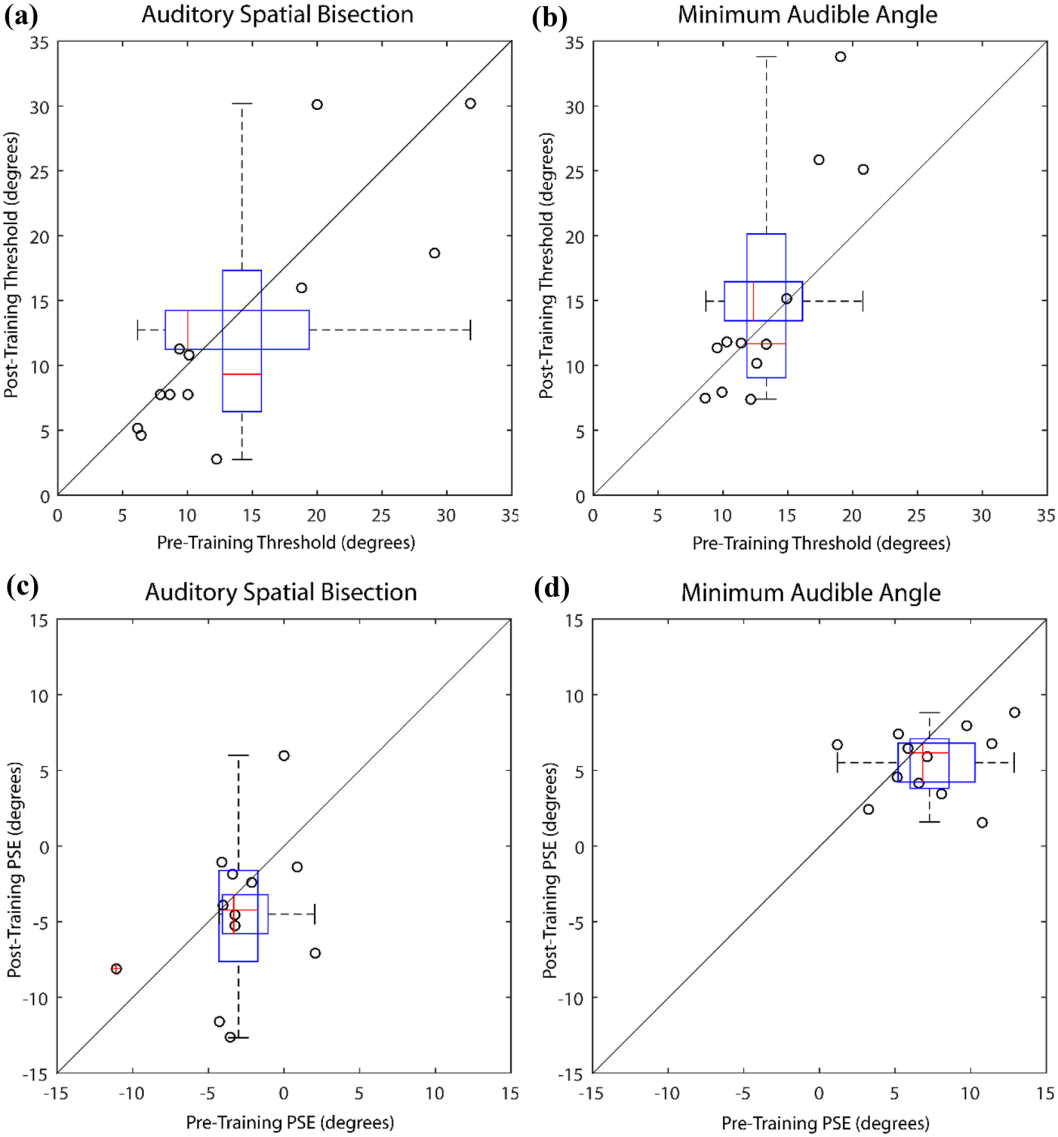
Performance of participants who were blind in auditory spatial bisection and minimum angle tasks before and after training. Sample size was 12 participants in all cases. Thresholds in **A** spatial bisection and **B** minimum angle tasks. Lower thresholds indicate better performance. PSE in **C** spatial bisection and **D** minimum angle tasks. Values closer to zero indicate lower response bias. In all plots circles represent data from individual participants, and boxplots data across participants. The plotted whiskers extend to values adjacent to 1.5 × the interquartile range, which is the most extreme data value that is not an outlier. 1.5 × IQR corresponds to approximately 99.3 coverage if data are normally distributed. Extreme values (outside of the 1.5 IQR range) are highlighted in red

Figure 3 shows threshold and PSE for all tasks and conditions.

For thresholds in spatial bisection tasks (Fig. 3A) there was no significant difference between performance in pre- vs post sessions (*t*(11) = 0.971; *p* = 0.352; correlation: 0.836; mean difference: 1.46; SD 5.21), and the estimated Bayes factor of 3.022 suggests that the data were 3.022 times more likely to occur under the null hypothesis that there was no difference between sessions. For thresholds in minimum audible angle tasks (Fig. 3B) there was no significant difference between performance in pre- vs post sessions (*t*(11) =  − 1.039; *p* = 0.321; correlation: 0.890; mean difference: − 1.61; SD 5.38), and the estimated Bayes factor of 2.848 suggests that the data were 2.848 times more likely to occur under the null hypothesis that there was no difference between sessions. For PSE in spatial bisection tasks (Fig. 3C) there was no significant difference between performance in pre- vs post sessions (*t*(11) = 1.055; *p* = 0.314; correlation: 0.384; mean difference: 1.48; SD 4.86), and the estimated Bayes factor of 2.807 suggests that the data were 2.807 times more likely to occur under the null hypothesis that there was no difference between sessions. For PSE in minimum audible angle tasks (Fig. 3D) there was no significant difference between performance in pre- vs post sessions (*t*(11) = 1.631; *p* = 0.131; correlation: 0.196; mean difference: 1.76; SD 3.74), and the estimated Bayes factor of 1.496 suggests that the data were 1.496 times more likely to occur under the null hypothesis that there was no difference between sessions. We also tested for a significant change in proportion correct (including only those on trials on which there was an objectively correct answer), and found no evidence of any change in participants’ accuracy with this measure either in the spatial bisection task (*t*(11) = 0.050, *p* = 0.961) or minimum audible angle task (*t*(11) = 0.713, *p* = 0.491).

Although there had been no significant changes on the group level, we further investigated if any idiosyncratic changes in acoustic spatial abilities between pre and post sessions were correlated with outcomes in echolocation training. Specifically, for each participant we calculated their difference in performance in terms of threshold and PSE between pre and post measurement in acoustic spatial abilities (leading to 4 data points for each participant) and correlated this with their improvement between the first and last session in any of the measures used in any of our training tasks (seven data points for each participant; these data are also contained in the Supplementary Material S1). We then correlated these with one another, i.e. we ran a total of 4 × 7 = 28 correlation analyses. Although there were two positive correlations [i.e. correlation between change in participants point of subjective equality (PSE) for the MAA task and their improvement in the distance at which they echolocated object orientation was *r* = 0.608; *p* = 0.036 (uncorrected), and correlation between change in participants PSE for the Spatial Bisection task and their improvement in the distance at which they echolocated object orientation was *r* = 0.642; *p* = 0.025 (uncorrected)], these results did not survive correction for multiple comparisons. In sum, our data do not suggest a relationship between idiosyncratic changes in acoustic spatial abilities assessed in this experiment with echolocation training outcomes.

Thus, although previous research has shown that blind echolocation experts performed better than blind participants on both of these tasks of auditory localization (Tonelli et al. 2020; Vercillo et al. 2015), we did not find evidence supporting the idea that performance improved with echolocation training in blind people. It is important to note that this null effect is not due to a limited ability of participants to learn click-based echolocation—in fact, participants’ performance in click-based echolocation improved in three different echolocation tasks (size discrimination, orientation identification, virtual navigation) to a level that in most (but not all) cases matched performance demonstrated by experts (Norman et al. 2021; and Supplementary Material S1). Thus, significant improvement in click-based echolocation ability over the course of 10 weeks was not sufficient to confer improvements in auditory localization ability.

## Discussion

Blindness has been shown to be associated with impairments on some spatial hearing tasks (Zwiers et al. 2001; Lewald 2002; Gori et al. 2014), leading to the belief that visual experience plays a fundamental role in the calibration of mental representations of space. There is, however, some correlational evidence that blind people with expertise in click-based echolocation (a non-visual sensory skill) do not show such impairments and, in fact, show superior abilities (Vercillo et al. 2015; Tonelli et al. 2020). This highlights the possibility that echolocation might perform a similar role to vision in the calibration of space. In this study, we tested the causal nature of this association using a 10-week training program in click-based echolocation. We found over the course of our 10-week training program, however, that there was no evidence of improvements on performance in spatial hearing tasks (either minimum audible angle or spatial bisection of sound sources). This was the case despite participants showing substantial improvements in echolocation ability, often to a level that was comparable to that of expert echolocators. Thus, we conclude that improvements in echolocation ability are not sufficient to bring about improvements in the calibration of auditory space.

One explanation for why we did not observe training effects is that the effects of training in click-based echolocation on spatial perception relevant to these tasks may take a longer time to develop. Experts in previous studies had used click-based echolocation over many years, even decades. Thus, a 10-week period might not be long enough for the effects to take place. Related to this, even though training led to a dramatic improvement in echolocation ability in all participants, highest performance was achieved only at the end, and performance did not match the performance of experts in all tasks [e.g., in the size discrimination task, participants did not perform as well as experts even after 10 weeks of training, see Norman et al (2021)]. Thus, it is possible that more training or longer periods of use of this skill might be needed to achieve effects.

Importantly, comparing performance of our participants in the bisection task to performance of participants who were blind reported in Vercillo et al (2015) and Gori et al (2014) showed that on average our group of blind participants performed comparable to participants in those studies, both with respect to threshold and with respect to PSE. Furthermore, we observed a wide spread of performance in our sample. Thus, it is unlikely that our study is limited by ceiling or floor effects. Power analysis also shows that our sample size was adequate to detect hypothesized effects.

In conclusion, at this point our findings in combination with previous reports (Tonelli et al. 2020; Vercillo et al. 2015) neither support nor refute the idea that click-based echolocation may replace the role played by visual sensory calibration. Click-based echolocation has benefits for people who are blind in terms of mobility, independence and wellbeing (Norman et al. 2021; Thaler 2013). Thus, based on the practical relevance of this skill for people with vision impairments, we strongly suggest that future research is needed to determine if training in click-based echolocation over longer periods of time may improve auditory localization skills in people who are blind, and to identify if other factors can explain the association between echolocation expertise and superior auditory localization that have been reported previously (Tonelli et al. 2020; Vercillo et al. 2015).

## Supplementary Information

Below is the link to the electronic supplementary material.

Supplementary Material S1. Excel table containing data from all participants in all tasks and measurements. Each row contains data for one participant. Column headings indicate which data are shown. Columns A-C show participant information, columns D-C show data from the current experiment, and columns L-GH show echolocation training data. Details of the echolocation training and training results are described in Norman et al (2021) (XLSX 25 kb)
